# P-1906. From Hallway Chats to Best Practice: Mapping Gaps in Curbside Etiquette and Education

**DOI:** 10.1093/ofid/ofaf695.2075

**Published:** 2026-01-11

**Authors:** Matthew Gwiazdon, Wendy Stead

**Affiliations:** Beth Israel Lahey Health (Plymouth), Plymouth, MA; Beth Israel Deaconess Medical Center, Boston, MA

## Abstract

**Background:**

Informal consults, referred to as “curbsides,” are ubiquitous in modern medicine. A curbside occurs when a provider asks a question of a specialist that is answered without seeing the patient. Nuances vary by institution and specialty. In the absence of consensus on best practices, the process of curbsiding is typically informally taught, often by modeling. We sought to characterize attitudes and expectations for curbsides amongst internal medicine (IM) and infectious diseases (ID) providers to inform future formal education to optimize this practice.

**Figure 1.**
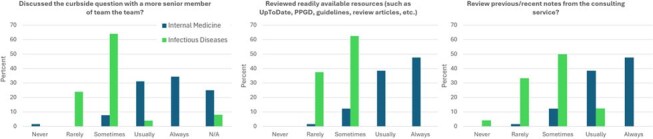
IM and ID providers’ estimations of the frequency with which primary teams review various available resources prior to engaging consultants with curbside questions. IM providers were asked to estimate the frequency of their own behaviors whereas ID providers were asked their perception of primary teams’ behaviors. The option of N/A (not applicable) was available to IM providers when asked about discussing the question with a more senior member of their team given the question’s lack of relevance to attending physicians.

**Figure 2.**
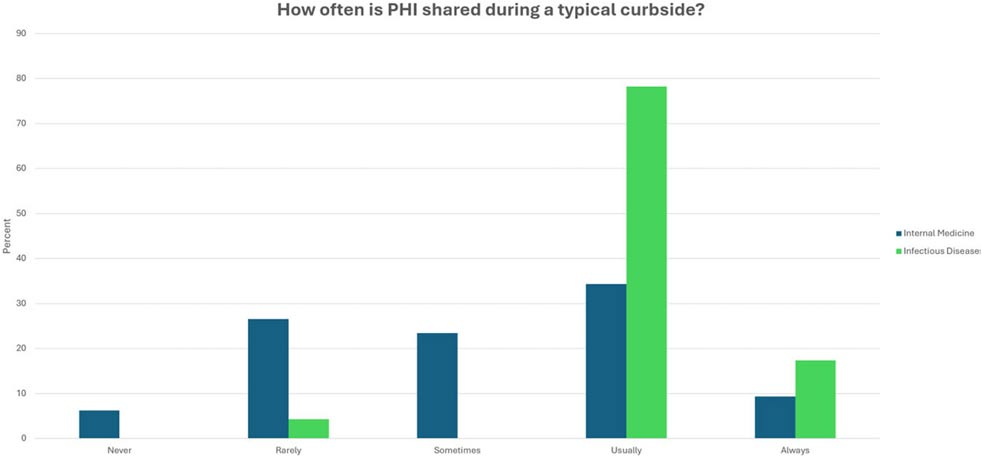
IM and ID providers’ estimations of how often patient specifics/PHI are shared with consultants during a typical curbside interaction. PHI: protected health information.

**Methods:**

Two online anonymous parallel surveys were sent to IM and ID trainee and faculty providers at an urban academic medical center regarding perceptions about current curbside practices.

**Figure 3.**
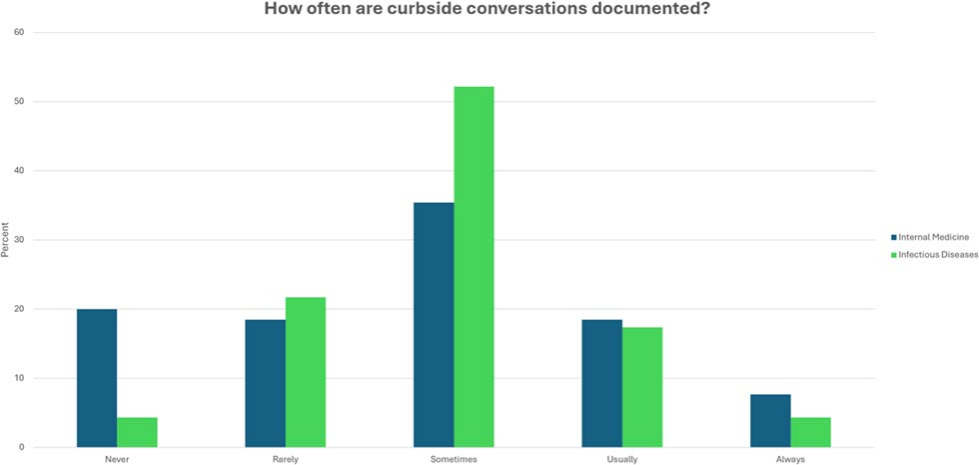
IM and ID providers’ estimations of the frequency with which informal curbside conversations are documented in the electronic medical record.

**Table 1.**
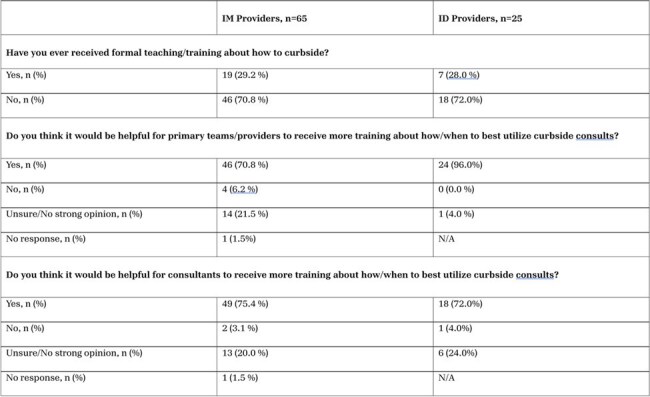
Survey responses from both IM and ID providers when asked whether they had received formal curbside education and whether or not they thought it would be helpful for primary providers and consultants to receive additional training.

**Results:**

90/398 providers responded to our surveys for an overall response rate of 22.6%. While IM providers endorsed reviewing important, case-relevant resources before asking a curbside question, ID providers perceived this as occurring less reliably (Figure 1). 95.7% of ID respondents indicated that protected health information (PHI) is Usually or Always shared during curbsides compared with only 43.8% of IM providers (Figure 2). 61.6% of IM and 73.9% of ID providers indicated that informal curbside recommendations are often formally documented in the medical record (Figure 3). More than 70% of IM and ID providers reported never having formal education about how to curbside and a similar portion of both groups believe both parties would benefit from additional training (Table 1).

**Conclusion:**

In the absence of formal guidance, expectations for optimal curbside conduct vary between IM providers and ID consultants. Despite the informal nature of curbsides, specific patient PHI is often shared and curbsides are often documented formally in the medical record. Given the ubiquitous practice and value of curbside interactions at academic medical centers, education about curbsiding should not be relegated to the “hidden curriculum.” A majority of consulting IM providers and ID consultants desire formal training on this topic. Insights from our survey can inform future curricular interventions on this important topic.

**Disclosures:**

All Authors: No reported disclosures

